# Trance and Possession Disorder With Underlying Dysthymia: A Case Report

**DOI:** 10.7759/cureus.54991

**Published:** 2024-02-26

**Authors:** Yatika Chadha, Ragini Patil, Saket Toshniwal, Nayan Sinha

**Affiliations:** 1 Psychiatry, Jawaharlal Nehru Medical College, Datta Meghe Institute of Higher Education and Research, Wardha, IND; 2 Medicine, Jawaharlal Nehru Medical College, Datta Meghe Institute of Higher Education and Research, Wardha, IND

**Keywords:** mental health, possessional state, dysthymia, mood disorder, trance and possession disorder

## Abstract

Trance and possession disorder (TPD) is an intriguing and complex phenomenon in the realm of psychology and psychiatry. Trance is characterized by a state of temporary marked alteration in the state of consciousness without replacement by an alternate identity, with either a narrowing of awareness of immediate surroundings or behaviors that are beyond one’s control. Possession is defined as an episode of alteration in the state of consciousness with the replacement of the customary sense of personal identity by a new identity, identified by the patient or his entourage as the spirit of an animal, a deceased individual, a deity, or a power. This often manifests culturally and contextually, varying in intensity and duration across different societies and belief systems, which could be due to an interplay of emotional stress and repressed emotions, domestic discord, or sociocultural issues. We report a case from Maharashtra, India, involving a patient diagnosed with TPD with underlying dysthymia. This case also highlights the complex interplay between these two psychiatric conditions and how managing one condition subsequently ceased the trance episodes.

## Introduction

Trance and possession disorder (TPD) is a rare and complex psychiatric condition characterized by episodes of altered consciousness, during which individuals may exhibit behavior, speech, or actions inconsistent with their usual personality. These episodes often resemble a state of trance or possession and can be challenging to diagnose and manage. Possession is a broad folk term used to explain a variety of symptoms or problems [1]. An extreme narrowing or total loss of awareness of one's immediate surroundings, which manifests as a significant lack of responsiveness or sensitivity to environmental stimuli, is the defining feature of this condition.

In addition to transient paralysis or loss of consciousness, the unresponsiveness may be accompanied by certain stereotyped actions (such as finger movements) that the individual is unaware of and/or is unable to control. There is no widely accepted collective cultural or religious practice that includes the dissociative trance [2]. It is frequently associated with possession-form presentations, marked by talking in a different voice, shaking, glossolalia, or making animal sounds, or “night dances” where the person exhibits altered states of consciousness coupled with rhythmic movements and convulsions; these dances are associated with rituals or ceremonies aimed at summoning spiritual forces [3]. In the International Classification of Diseases 11th Revision (ICD-11), the diagnosis is labeled as possession trance disorder (PTD). This acknowledges the cultural significance of possession experiences and their impact on individuals within specific cultural contexts [4]. Dysthymia, a chronic form of depression characterized by depressed mood most of the day and lasting for at least two years, may contribute to the development or exacerbation of TPD symptoms.

## Case presentation

A 55-year-old married female with two children, a homemaker by occupation from a lower socioeconomic class without any formal education, was brought in by her daughter, with complaints of regular headaches, low mood, difficulty performing daily tasks, sleep difficulties, and withdrawn behavior. The daughter reported that she had been acting strangely, especially on full moon nights, when she would experience episodes where she would act differently and speak in a different voice, sit unusually still, and experience bouts of crying. The patient reported that she could only partially recall these episodes and that she frequently experienced episodes of altered consciousness, unexplainable behavior, and feeling "possessed". The patient’s episodes typically occurred without any warning and lasted for approximately 30 minutes to two hours. These episodes had been happening for seven years and the patient, from a lower socioeconomic strata, had only been cared for by faith healers primarily.

**Figure 1:**
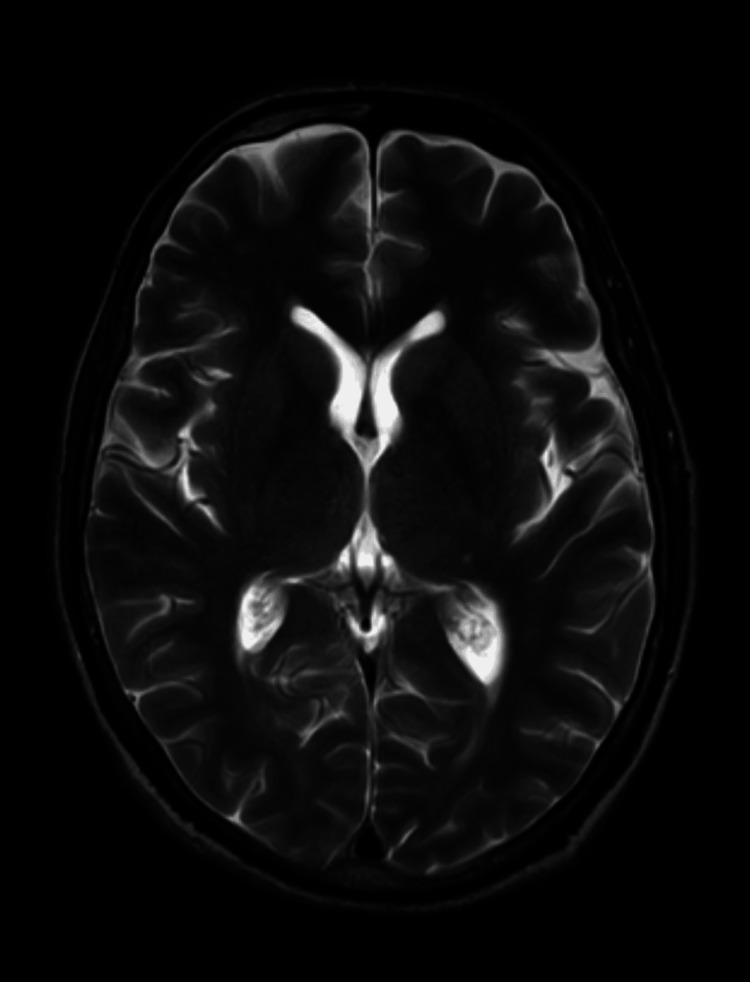
MRI - T2-weighted axial section of the brain suggesting normal imaging study MRI: magnetic resonance imaging

To establish an accurate diagnosis, it was necessary to rule out any organicity, and hence routine investigations such as complete blood count and liver and kidney function tests were performed, which came within normal limits. A comprehensive physical and neurological examination was also done, which was unremarkable. A drug panel could not be done as the patient could not afford it; however, there was no history of drug use/abuse, which was corroborated by a reliable informant as well. A comprehensive medical evaluation was done, including an electroencephalogram (EEG) to rule out any complex partial seizure-like episode. Also, an MRI of the brain was performed to rule out any central nervous system lesions or a potential tumor that could lead to TPD possession disorder (Figure 1).

The patient had a history of recurrent depressive symptoms dating back to her adolescence, which she had taken for several years with wavering compliance and therefore with limited improvement in her mood symptoms. Her depressive symptoms included low energy, feelings of hopelessness, and social withdrawal. To summarize, the patient exhibited altered consciousness, speaking in a different voice or language, expressing intense emotions and often crying, and making statements that were inconsistent with her personality and beliefs.

## Discussion

TPD cases are mostly reported in Asian countries, with limited cases reported from India due to the sociocultural stigma attached to this condition as well as other mental health disorders. The first episode of TPD is reported to have occurred around 25 years of age. It has been reported that the possessing agent is well known, where it either corresponds to a well-known entity, associated with the culture of the patient/universal figure of god or devil [5]. Trance episodes were reported among female coworkers of mentally ill people who were found visiting a temple in India (Maharashtra) where they described these episodes as a way of directing pain away from the person who is experiencing it [6].

The timeframe of possession trance episodes can vary widely, with episodes occurring intermittently or in response to specific triggers, such as religious or spiritual ceremonies, emotional distress, or cultural events. These episodes may last minutes to hours and can be recurring over time [6]. This report describes a case of TPD in Maharashtra and emphasizes the importance of recognizing the co-occurrence of TPD with underlying dysthymia. The successful management of our patient's condition highlights the potential benefits of a holistic treatment approach that addresses both TPD with the associated mood disorder, thereby helping the patient regain their normal selves.

TPD is not recognized as a distinct diagnosis in the Diagnostic and Statistical Manual of Mental Disorders Fifth Edition (DSM-5); instead, these states are categorized under other mental health disorders, cultural syndromes, or dissociative trance, which comes under dissociative disorders and only applies to those who experience an unusual involuntary event; neither a regular activity nor a normal experience will qualify, according to DSM-5, which states that the patient must fulfill the following criterion: disruption of identity and altered consciousness to the surroundings that may be perceived as an experience of being controlled by an external supernatural power [7,8]. It affects both sexes equally, and a higher incidence of possession, as opposed to trance, was observed to be accompanied by hallucinatory symptoms during episodes, meaning that this should be considered a significant factor and evaluated [9]. TPD manifests in many forms, and one study has reported a pseudo-seizure episode that later turned out to be a trance state, highlighting the need for a multidisciplinary approach involving psychiatry for integrated and best possible medical care [10].

Our patient’s clinical presentation met the diagnostic criteria for TPD, and her underlying dysthymia was confirmed through a thorough psychiatric evaluation. It is crucial to evaluate possession trance experiences by considering if the symptoms might be better explained by substance or medicine abuse, a syndrome unique to a particular culture, or another mental illness. Various differentials along with a comparison of their clinical and cultural presentations are presented in Table 1.

**Table 1:** Differential diagnosis and comparison of their clinical and cultural presentations with TPD TPD: trance and possession disorder

	Trance and possession disorder (TPD)	Dissociative identity disorder (DID)	Psychotic disorders
Cultural and religious context	Present	Not inherently linked	Not inherently linked
Presence of altered identities	Involves a perceived influence or control by external entities or spirits	Presence of two or more distinct personality states	Includes hallucinations, delusions, disorganized thinking, and grossly disorganized or abnormal motor behavior
Response to cultural context	Associated within the cultural and religious framework and may be viewed as normative or spiritually significant	Considered a psychiatric condition with a focus on identity disturbances and amnesia	Often treated from a clinical perspective, with less emphasis on cultural or religious interpretations

However, these differentials were ruled out in our case as the patient had no history of substance use/abuse, which was corroborated by a reliable informant. This warranted eliminating any other possible explanation for the symptoms, such as underlying medical issues, drug-induced states, or other mental health issues. Episodes of possession trance can be so disruptive that they impair social and occupational functioning. People could find it difficult to focus on their work, fulfill social obligations, and go about their everyday lives both during and after these episodes [2]. Thereafter, a treatment plan was formulated, which included an antidepressant medication to manage dysthymia with tablet escitalopram 10 mg (one tablet at night for the initial week of treatment for sleep disturbances, which was later tapered). Also, weekly individual psychotherapy sessions were planned to address depressive symptoms, improve coping strategies, and explore underlying emotional conflicts; regular psychiatric follow-ups were scheduled to monitor progress and implement adjustments to treatment as needed.

Over the next several months, the patient showed gradual improvement in her depressive symptoms, and a marked reduction in the frequency and intensity of trance episodes was observed. Psychotherapy played a crucial role in helping her understand the link between her depressive tendencies and trance episodes. She learned effective coping strategies and assertiveness skills to manage stress and emotional triggers, which contributed to her overall well-being. The delay in seeking treatment for TPD, secondary to stigma (mainly observed in rural areas), can worsen the patient's symptoms [11,12]. The culture and beliefs of the patient's family, and fostering a positive attitude in the community toward such mental disorders play a major role in the treatment of these patients [13].

## Conclusions

Patients with TPD require a thorough and individualized treatment strategy that incorporates psychotherapy, medication, and specialized therapies, especially when the disorder is comorbid with dysthymia. If our patient's prior psychiatric diagnosis of dysthymia had been recognized and treated appropriately, the trance episodes may not have occurred in the first place. Diagnosing TPD poses several challenges and limitations due to the complex interplay of cultural, spiritual, psychiatric, and socioeconomic factors. Therefore, early diagnosis and treatment are crucial to enhance the patient's general functioning and quality of life. Additionally, it is important to raise awareness about such cases among the general public as well as among medical professionals to facilitate a quick diagnosis and management.
